# Effects of dietary supplementation in treatment and control of progression and complications of insulin-dependent diabetes mellitus: a systematic review with meta-analyses of randomized clinical trials

**DOI:** 10.1590/1414-431X2024e13649

**Published:** 2024-08-23

**Authors:** L.C. Ferraz, M.D.R. Barros, K.M.M. Almeida, M.B.G. Silva, N.B. Bueno

**Affiliations:** 1 Laboratório de Nutrição e Metabolismo, Faculdade de Nutrição, Universidade Federal de Alagoas, Maceió, AL, Brasil

**Keywords:** Diabetes complications, Endocrine system diseases, Glycemic control, Biomarkers, Nutritional therapy

## Abstract

There is no safe and effective prevention for insulin-dependent diabetes (IDDM) mellitus, which makes it highly dependent on its treatment. This systematic review with meta-analyses of randomized clinical trials investigated the overall effects of dietary supplements of vitamins, minerals, trace elements, and non-essential compounds with antioxidant properties, fatty acids, and amino acids in IDDM. Searches of MEDLINE, Embase, CENTRAL, LILACS, The Grey Literature Report, and ClinicaTrials.gov, and citations from previous reviews were used to identify reports published through July 2023. The Risk of Bias 2 (RoB2) tool was used to analyze the risk of bias and GRADE was used to assess the quality of the results. Fifty-eight studies (n=3,044) were included in qualitative analyses and seventeen (n=723) in meta-analyses. Qualitative analyses showed few positive effects on some metabolic function markers, such as endothelial and renal function and lipid profile. Meta-analyses showed a positive effect of omega-3 on glycated hemoglobin (HbA1c) (RMD=-0.33; 95%CI: -0.53, -0.12, P=0.002; I^2^=0%; GRADE: low quality; 4 studies) and of vitamin D on fasting C-peptide (FCP) (RMD=0.05; 95%CI: 0.01, 0.9, P=0.023; I^2^=0%; GRADE: very low quality; 4 studies). Most studies showed bias concern or high risk of bias. A recommendation for dietary supplementation in IDDM cannot be made because of the few positive results within different interventions and markers, the serious risk of bias in the included studies, and the low quality of evidence from meta-analyses. The positive result of vitamin D on FCP is preliminary, requiring further investigation.

## Introduction

Insulin-dependent diabetes mellitus (IDDM) is one of the types of diabetes mellitus (DM) that accounts for up to 10% of all cases of DM in adults, with the most common onset in children and adolescents (1). The global prevalence is about 1.21 million patients younger than 20 years, with an incidence of 149,000 new cases annually in this age group (2). The autoimmune etiology is present in 90% of cases, with the destruction of pancreatic β-cells, which are responsible for producing insulin, in a process of apoptosis via humoral and cellular autoimmunity induced by inflammatory reactions with high levels of cytokines (3). As a result, the body is unable to produce this hormone and there is a consequent increase in blood glucose, since insulin reduces blood glucose by signaling the translocation of GLUT4 in the membranes of cells that capture glucose (4). It is a chronic disease characterized by increased blood glucose, which can lead to damage to various organs and systems if long-term uncontrolled high plasma glucose levels persist for a long period of time. Early discovery and appropriate treatment management are the best way to delay or even prevent the onset of these complications (2,5).

Despite the progress of research to better understand IDDM, there is still no safe and effective way for its prevention (3), making it a disease highly dependent on its treatment, which is mainly based on the administration of exogenous insulin, making it a costly therapy with limited access, in addition to dietary control and exercise (4). Nutritional treatment aims at glycemic control, with studies and nutritional recommendations focusing on the distribution of macronutrients, especially the consumption of carbohydrates, in addition to the carbohydrate counting technique, which optimizes the use of insulin according to the amount of ingested carbohydrate (6).

There is no recommendation for the intake of micronutrients or specific compounds other than values recommended for the healthy population (6). The need to adjust micronutrients in type 1 diabetic patients is crucial, because their deficiency and low consumption has already been reported in patients with uncontrolled diabetes, who are more susceptible due to high levels of oxidative stress and inflammation, which can increase the demand for micronutrients (7-9). Despite this, only reviews without quantitative analysis and only using vitamin D supplementation have been carried out on IDDM (10,11).

Hence, this systematic review with meta-analyses aimed to compile the results of randomized clinical trials (RCTs) with nutritional supplementation interventions, especially of vitamins, minerals, trace elements, non-essential compounds with antioxidant properties, fatty acids, and amino acids, to summarize the effects of these interventions in individuals with IDDM, guiding intake recommendations and future research on this topic.

## Material and Methods

### Data availability and transparency statement

This research followed the recommendations of the Cochrane Collaboration Handbook for Systematic Reviews of Interventions (12) and is reported according to Preferred Reporting Items for Systematic Reviews and Meta-analyses PRISMA 2020 (13) (Supplementary Figure S1). The protocol was published and approved by the International Prospective Register of Systematic Reviews (PROSPERO) under the registration code CRD42021266766.

### Search strategies

Searches were performed in the main electronic bibliographic databases MEDLINE, EMBASE, Cochrane Central Register of Controlled Trials (CENTRAL), in the Latin American regional database LILACS, in the Gray Literature database Gray Literature Report (https://www.greylit.org), and in the Clinical Trials Registry ClinicalTrials.gov. In addition, studies cited by previous systematic reviews on this topic were manually included for screening. For the search strategy in the main databases, the following terms were used: “insulin-dependent diabetes mellitus”, “vitamin”, “trace element”, “mineral”, “antioxidant”, “amino acid”, and “fatty acid”, with slight changes for the other databases (Supplementary Table S1).

In the MEDLINE database, a highly sensitive strategy to identify RCTs proposed by Cochrane was used (12). For the Embase and CENTRAL databases, the filters of the platforms were used to identify this type of study, while in ClinicalTrials.gov, a filter was used to locate only studies with results. For LILACS, filters for RCTs were not used due to the low number of reports found and to avoid the loss of possible eligible studies. The searches had no restrictions on time or language of publication, and the last update was performed in July 2023.

### Eligibility assessment

This study included RCTs with two groups of people with IDDM with or without other associated diseases: one group receiving oral dietary supplements of vitamins, minerals, trace elements, non-essential compounds with antioxidant properties, amino acids, and fatty acids and one group receiving a placebo supplementation. The supplemented compounds needed to be in the natural form or as corresponding synthetic metabolites, have a defined concentration, and be taken alone or combined with another substance of the same or different nature. Individuals with other DM types, food extracts, except for concentrated oils used as fatty acid supplementation (e.g., fish oil), and compounds associated with medications as an intervention were excluded.

Furthermore, other dietary supplements or therapies and studies with subgroups diagnosed with IDDM that did not present the results separately from other subgroups were also excluded. There was no restriction on age, sex, and ethnicity of the participants of the studies.

### Data extraction

Two independent reviewers were involved in study selection, data extraction, risk of bias assessment, and quality of evidence assessment, following the Cochrane guidelines for systematic reviews (12), acting in parallel in all stages of the selection of studies eligible for inclusion and data extraction, with a third independent researcher available to resolve conflicts between the two reviewers. Relevant studies identified by title and abstract screening were selected for full-text review and included or excluded according to the eligibility criteria. Qualitative and quantitative data from the final reports were compiled into tables in Excel software (Microsoft, USA) for further organization and analyses. The following data were extracted from each selected trial: first author, publication year, location (country), study design, duration of intervention, sex, number of participants, age of participants, intervention, dose and frequency of the intervention, main analyzed outcomes, and effect data (significant changes in intervention *vs* placebo values).

### Criteria for inclusion in the systematic review

Following the eligibility criteria, the remaining reports that analyzed the effects of supplementation on different biomarkers were included in the qualitative assessment of the data. The primary endpoints to this review were changes in glycated hemoglobin (HbA1c) and fasting glucose. Insulin requirement, fasting C-peptide (FCP), hypoglycemia events, regulatory T-lymphocyte activity, lipid profile, body composition, inflammatory cytokines, platelet reactivity, thromboxane production, flow-mediated dilation, residual β-cell activity markers, antioxidant profile, and disease progression endpoints (neurological, macrovascular, microvascular, and metabolic disorders) were secondary endpoints.

### Risk of bias and quality of evidence assessment

The Cochrane RCTs Risk of Bias tool version 2 (RoB 2) was used to assess the quality of the included studies. The risk of bias is assessed in 5 domains: randomization of participants, loss of participants from the intervention group, missing outcome data, measurement of outcomes, and selection of reported outcomes. An algorithmically generated result based on the answers to the specific questions is obtained, categorizing each domain, giving the study an overall rating of “low risk”, “high risk”, or “some concern” (14).

Grading of Recommendations, Assessment, Development, and Evaluation (GRADE) tool was used to assess the quality of evidence and the strength of health recommendations for the results obtained in the meta-analyses, classifying them into “very low”, “low”, “moderate”, or “high” quality of evidence on the basis of the design of the studies, risk of bias, inconsistency, indirect evidence, imprecision, and publication bias (15).

### Criteria for inclusion in the meta-analyses

Meta-analyses were performed on outcomes that were analyzed by three or more reports included on the systematic review when their data were presented in an extractable form, i.e. in mean and standard deviation or mean and standard error of the mean for both groups, with the standard deviation value converted from the sample data. Outcome data from studies with an adult, adolescent, or child sample were included, and studies with pregnant women were not.

### Statistical analyses

The analyses of the raw mean difference (RMD) were used when the data of the reports were presented in the same unit or units with direct conversion into percentage (e.g., HbA1c, in percentage (%) and mmol/mol; FCP, in ng/mL, pmol/mL, and nmol/L) for HbA1c and nmol/L for FCP. Standardized mean difference (SMD) analysis was used for outcomes with different measurement units without the possibility of direct conversion (e.g., insulin dose requirement, in IU/kg/day and IU/day).

The weights for each study were assigned using the inverse of variance, performing a random effects analysis by the DerSimonian & Laird method. Tau^2^ statistic was used to identify heterogeneity, considering P<0.10 as significant, and inconsistency was determined by the I^2^ statistic, considering ≤40% a low value (16). Subgroup, publication bias, and meta-regression analyses could not be performed due to the low number of studies included in each meta-analysis. All statistical analyses and graph plotting were performed in Jamovi software version 1.6.23 (The Jamovi Project, Australia).

## Results

### Characteristics of the included studies

**Figure 1:**
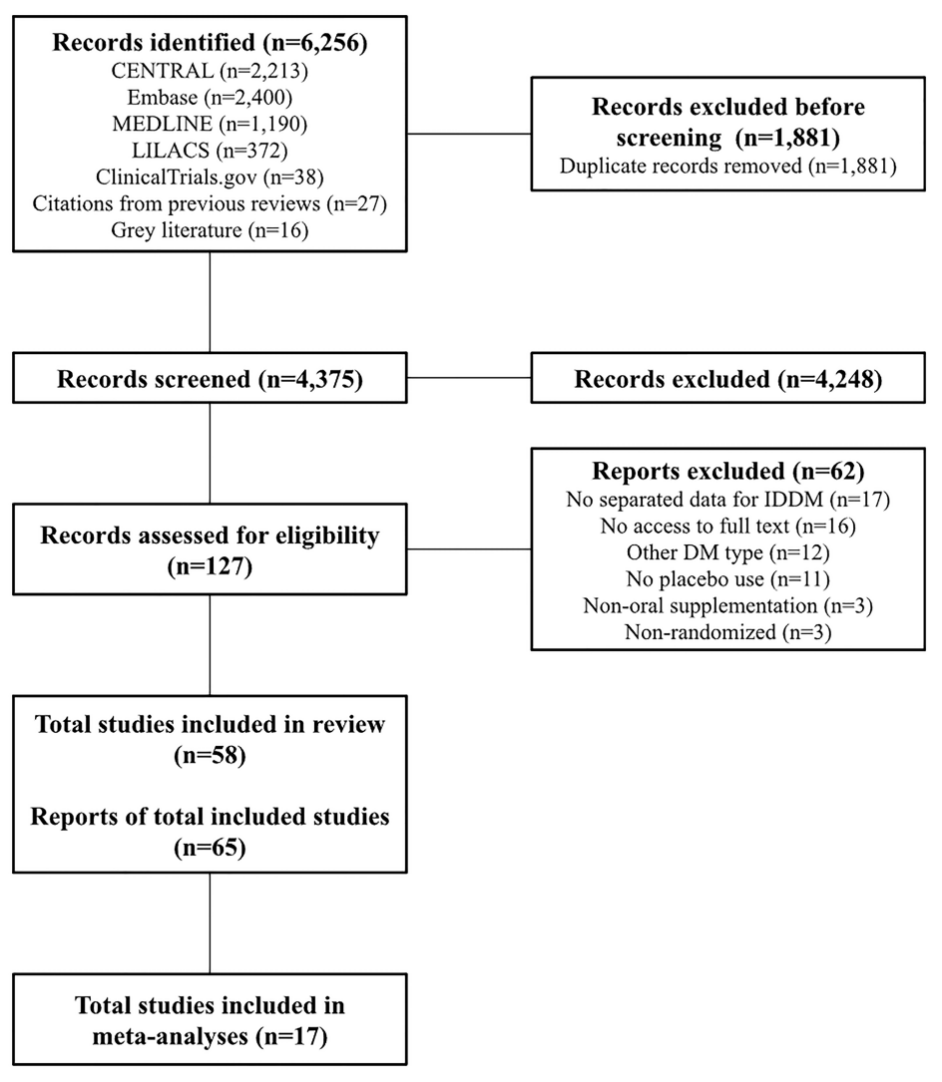
PRISMA 2020 flow diagram of the search and selection of included studies and reports.

The screening process started with 6,229 records in the electronic databases and 27 reports of systematic reviews related to the topic, totaling 6,256 records. At the end, 65 reports belonging to 58 studies were included in the systematic review, and 17 were included in the meta-analyses. All steps of the search process and selection of studies and reports included in the analyses are shown in Figure 1.

The detailed characteristics of all 65 reports analyzed are presented in Supplementary Tables S2-S4 (17-81). In total, 44 (75.9%) studies had a parallel design, and 14 (24.1%) had a crossover design. The studies included a total of 3,044 randomized subjects, with the adult population being the most common among samples. For the diabetes duration, 12 reports worked with the new-onset disease within a few days or months of diagnosis to observe the preservation of pancreatic function, glycemic control, insulin, initial symptoms, or immune response. The other 53 studies worked with chronically established IDDM, aiming to control metabolic changes and complications of the disease.

### Systematic review

#### Vitamin B complex and vitamin D

On the glycemic control variable Hb1Ac, a significant difference was only reported with nicotinamide alone in one of five reports with this vitamin (27) and with the combined supplementation of vitamin B1, B6, and B12 (19). Nwosu et al. (36) demonstrated a significant difference in the variable of insulin dose required per day in addition to A1c-adjusted insulin dose with vitamin D (ergocalciferol).

Significant improvement in flow-mediated dilation was shown with combined and isolated supplementation of vitamin B6 and folate for eight weeks (23). Another important marker for endothelial function, homocysteine production showed a significant reduction in the study by Elbarbary et al. (19) after supplementation of vitamins B1, B6, and B12.

Joergensen et al. (34) was the only report with vitamin D supplementation present in this review that analyzed renal aspects between these compounds, having significant results in reduction of estimated glomerular filtration rate and albuminuria. The supplementation of vitamins B1, B6, and B12 also showed a significant reduction in urinary albumin/creatinine ratio and cystatin-C (21).

Cholecalciferol (vitamin D3) supplementation may improve the ability to suppress regulatory T lymphocytes in reducing the proliferation of effector T lymphocytes (37), in addition to the increase of chemokine ligand 2 (38). Additionally, tumor necrosis factor-α was reduced in the study by Nwosu et al. (36) using ergocalciferol.

#### Vitamin C and vitamin E

Among the three reports with vitamin C supplementation included in this review, Juhl et al. (40) found a significant reduction in the transcapillary escape rate. With vitamin E, flow-mediated dilation was improved only in two reports (54,55), with no significant result on these markers in studies with joint vitamin C and vitamin E supplementation. Markers related to platelet activity and blood coagulation were analyzed by studies with vitamin E supplementation, with only a significant decrease in thromboxane B2 production in the report by Gisinger et al. (51).

Ascorbic acid as prophylactic pre-exercise supplementation decreased the production of free radicals and lipid hydroperoxides (39), being the only study with vitamin C that analyzed these aspects. Decreased low-density lipoprotein (LDL) oxidation was significant in two reports (50,55), and the malondialdehyde production significantly decreased in the study by Colette et al. (46). Also, with alpha-tocopherol, the decrease in lipid peroxides by a reduction in the production of thiobarbituric acid reactive substances was observed in the report by Engelen et al. (49). In a more specific analysis of different variables and haptoglobin genotypes, the report by Costacou et al. (44) found higher HDL-mediated cholesterol efflux in the group with the Hp 2-2 genotype, but with worsening HDL particle size in the Hp 1-1 genotype.

#### Minerals, trace elements, and antioxidant compounds

Jumaah et al. (62) observed a beneficial effect of the use of zinc, magnesium, and vitamin D on signs and symptoms of diabetes and diabetic ketoacidosis, and Shidfar et al. (64) reported an increase over placebo in apolipoprotein A-I and a decrease in the apolipoprotein B/apolipoprotein A-I ratio with zinc and vitamin A intervention. Mollo et al. (67) was the only study that reported a positive effect with antioxidant supplementation, finding increased clotting time in patients who received alpha-lipoic acid (ALA) compared to those who received a placebo, in addition to lower expression of CD41 and CD62P lymphocytes, indicators of platelet activation.

#### Amino acid and fatty acids

Mauras et al. (69) and Torres-Santiago et al. (71) found a higher occurrence of nocturnal hypoglycemia episodes in the group that received the treatment with glutamine compared to the placebo group, raising the hypothesis of a possible mechanism of increased insulin sensitivity by glutamine, which was not observed by the latter study, which also performed this analysis.

Reports by Horvaticek et al. (74) and Ivanisevic et al. (75) are part of the same study conducted with pregnant women supplemented with omega-3 that analyzed glycemic control, C-peptide, and insulin dose variations. The results showed a higher FCP in the third semester in the group that received the supplementation compared to the placebo, in addition to a lower concentration of glucose and higher concentration of cord C-peptide in newborns and a significantly lower value of the IR-HOMA 2 parameter in children of mothers who received omega-3 supplementation. Analyzing endothelial and coagulation function variables, Khorshidi et al. (76) reported significantly improved flow-mediated dilation with omega-3 supplementation for 12 weeks. Haines et al. (73) observed a significant reduction in thromboxane production and increased coagulation factor X and platelet aggregation.

The analysis of cognitive test performance during induced hypoglycemia was the central outcome of two studies included in this review, one with the supplementation of an amino acid mixture (70) and the other with medium-chain fatty acid (MCFA) (80). In both studies, the group that received the intervention had better results than the group that received the placebo for most of the tests. As for a change in lipid profile, Rossing et al. (81) reported a significant decrease in very low-density lipoprotein (VLDL) and triglycerides (TGL), but an increase in total cholesterol after 12 months of treatment. Mori et al. (77) reported favorable results in a 3-week intervention, with a significant decrease in HDL3 and TGL subfractions and an increase in HDL2 subfraction. A significant decrease in plasma triglyceride was also found by Khorshidi et al. (76), with no changes in total cholesterol and its fractions. Britten-Jones et al. (72) showed a neuroprotective effect of omega-3 confirmed by significant positive results in central corneal nerve fiber length, central corneal nerve branch density, and central corneal nerve fiber density.

### Meta-analyses

#### Effects of vitamin B3 supplementation

**Figure 2:**
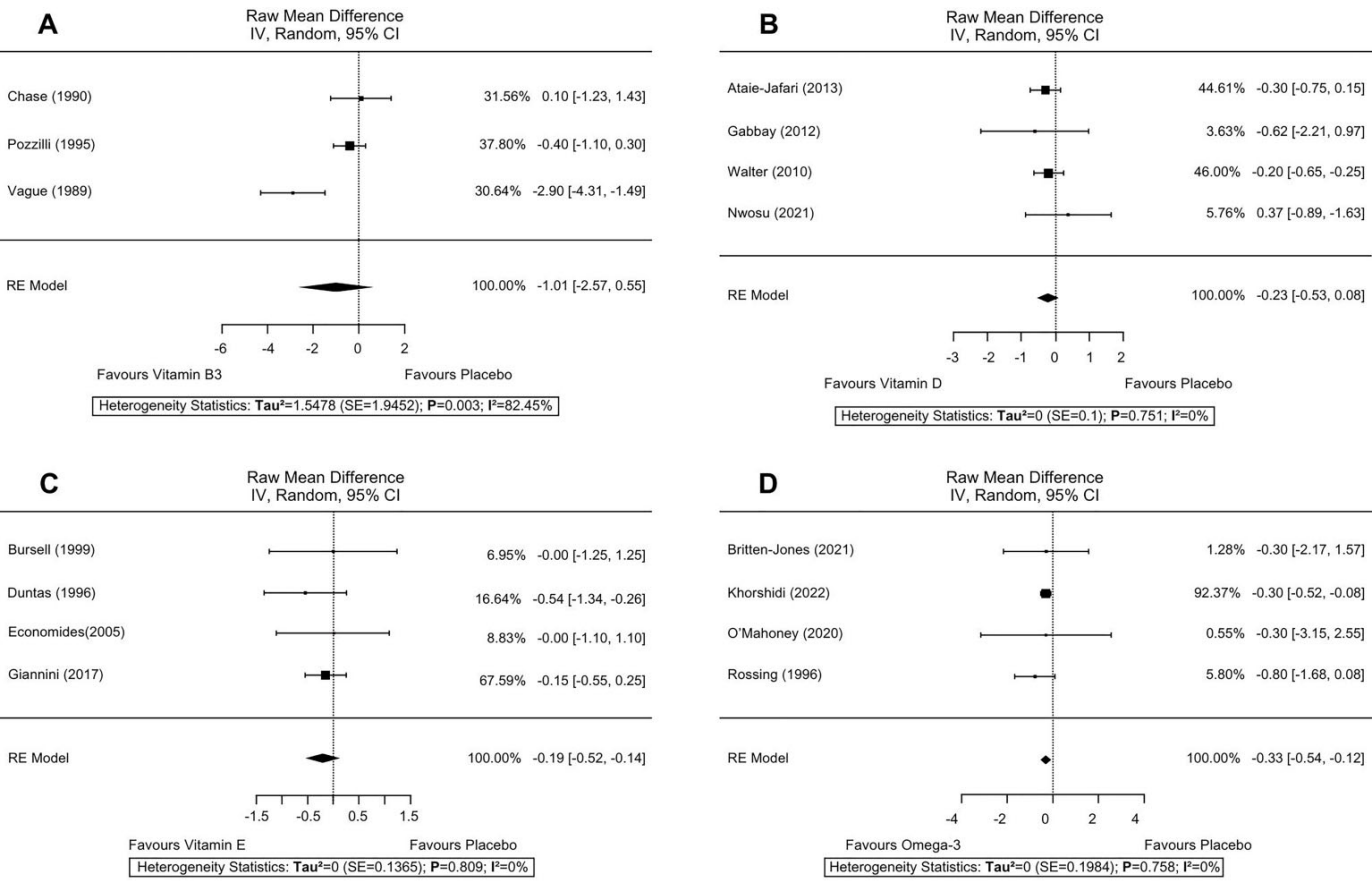
Effects of dietary supplementations on glycated hemoglobin (HbA1c). **A**, Vitamin B3; **B**, Vitamin D; **C**, Vitamin E; **D**, Omega-3.

**Figure 3:**
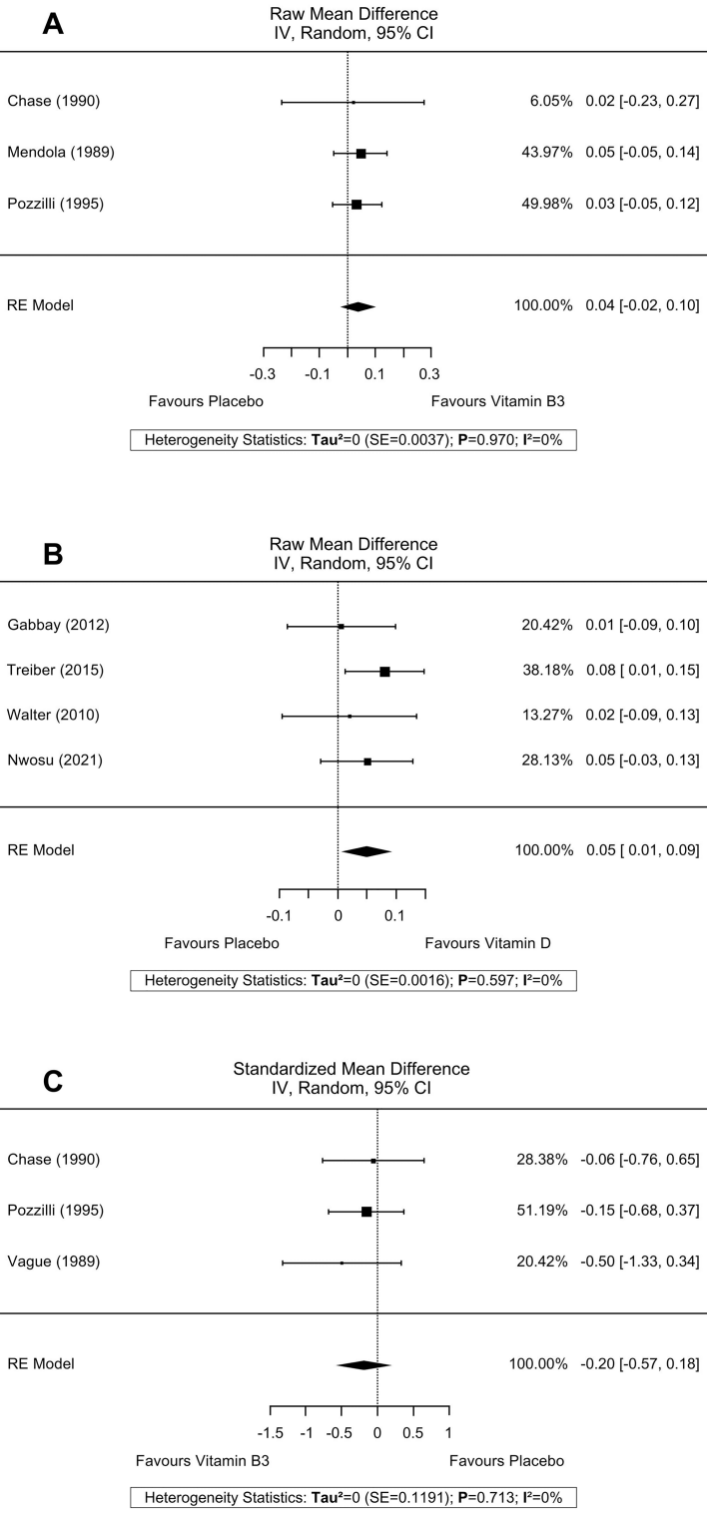
Effects of dietary supplementations on fasting C-peptide and insulin dose requirements. **A**, Vitamin B3 on fasting C-peptide; **B**, Vitamin D on fasting C-peptide; **C**, Vitamin B3 on insulin dose requirement.

Three reports (n=117) (18,26,27) with vitamin B3 supplementation had HbA1c data that was possible to extract for meta-analysis, showing a non-significant effect on improvement in glycated hemoglobin (RMD=-1.01; 95%CI: -2.57, 0.55; P=0.205), with high heterogeneity (I^2^=82.45%; P=0.003) (Figure 2A). Also, vitamin B3 supplementation (three reports, n=137) (18,22,26) showed no significant effect on FCP secretion (RMD=0.04; 95%CI: -0.02, 0.10; P=0.224), with low heterogeneity of data (I^2^=0%; P=0.970) (Figure 3A). Finally, there was a meta-analysis of data from three reports (n=117) (18,26,27) for the insulin dose required, which also showed no significant difference in results (SMD=-0.20; 95%CI: -0.57, 0.18; P=0.306) and low heterogeneity of the data (I^2^=0%; P=0.713) (Figure 3C).

#### Effects of vitamin D supplementation

Four reports (n=175) (30,33,36,38) with vitamin D supplementation on HbA1c showed no significant effect (RMD=-0.23; 95%CI: -0.53, 0.08; P=0.152), and low heterogeneity of data (I^2^=0%; P=0.751) (Figure 2B). However, FCP secretion data from four reports (n=144) (33,36-38) showed a C-peptide preservation effect with the intervention (RMD=0.05; 95%CI: 0.01, 0.9; P=0.023), with low data heterogeneity (I^2^=0%; P=0.391) (Figure 3B).

#### Effects of vitamin E supplementation

The only possible meta-analysis with vitamin E included four reports (n=113) (43,47,48,50) that presented HbA1c, demonstrating no significant effect with the intervention (RMD=-0.19; 95%CI: -0.52, 0.14; P=0.253), with low data heterogeneity (I^2^=0%; P=0.809) (Figure 2C).

#### Effects of omega-3 supplementation

Four reports (n=140) (72,76,79,81) were included for the meta-analysis of the effects of supplementation of omega-3 on HbA1c. The result showed a significant effect on HbA1c control (RMD=-0.33; 95%CI: -0.53, -0.12; P=0.002), presenting low heterogeneity in the data (I^2^=0%; P=0.758) (Figure 2D).

### Risk of bias analysis

The overall risk of bias assessment of each included study is indicated in the final column of the respective tables with the interventions in this review (Supplementary Tables S2-S4). Altogether, 16 studies were classified as high risk of bias, 32 as some concern for risk of bias, and 10 as low risk of bias, with deviations from intended interventions and selection of the reported outcome being the leading causes of bias in these reports (Supplementary Figure S2).

### Analysis of the overall quality of the evidence

The markers analyzed in the meta-analyses were defined as critically important (HbA1c) and important (FCP and required insulin dose) due to their direct relationship with the prognosis of IDDM. The meta-analyses were assessed as low or very low quality, with the serious risk of bias of the included studies being primarily responsible for the low quality of the results of the analyses, in addition to serious inconsistency, indirect evidence, and imprecision of some of them. Supplementary Table S5 shows the GRADE summary of findings for quality of evidence assessment for the effects of nutritional compounds supplementation in individuals with IDDM.

## Discussion

This extensive systematic review with meta-analyses of intervention studies with supplementation of vitamins, minerals, trace elements, and non-essential compounds with antioxidant properties, amino acids, and fatty acids showed inconsistent results among most of the outcomes analyzed. The primary endpoint of this review, variables of glycemic control, had only a few isolated positive significant results in the qualitative analysis of reports. Instead of glycemic control, most of the included studies had as central endpoint markers of other biological functions that undergo changes throughout the disease, such as endothelial, renal, and antioxidant defense, which were the secondary endpoints analyzed in this review. However, as with the markers of glucose metabolism, it was only possible to observe significant effects on isolated variables and rarely with positive results in more than one study with the same intervention.

Nicotinamide was one of the interventions in studies that looked at the effect of these compounds in preserving pancreatic β-cell function in newly diagnosed individuals with IDDM, with the hypothesis of the action being the inhibition of poly (ADP-ribose) synthesis, an enzyme involved in the process of cell apoptosis and other mechanisms (82). However, no effect was observed in the meta-analyses performed by this review on the preservation of C-peptide secretion or reduction of the required daily insulin dose (Figure 3), which are β-cell function markers.

In the same spectrum, the use of vitamin D supplementation in this context arises after the advancement of studies showing several interactions of the vitamin with different cells of the body, especially the immune system (83). The qualitative analysis of vitamin D studies showed significant changes in immunological markers, glycemic control, and inflammatory markers (33,36,37). Additionally, the meta-analysis on the secretion of FCP with the use of vitamin D showed a statistically significant result, which would indicate a protective action of vitamin D in controlling the progression of IDDM in its early stages, in line with the results of meta-analyses of observational studies that suggest high circulating 25(OH)D concentration and vitamin D supplementation in early childhood protect against the development of IDDM (84).

However, caution should be taken regarding the interpretation of these data before recommending vitamin D supplementation for the control of IDDM progression, primarily because this meta-analysis only included four studies with small samples and was rated by GRADE as ‘very-low' quality of evidence. Among the four studies included, three different formulations of vitamin D were used: calcitriol (38), ergocalciferol (36), and cholecalciferol (33,37), which have different metabolisms, so further research is needed to define the effects of vitamin D.

Vitamins of the B complex are related to endothelial function, and the use of folic acid has already been tested in other conditions, such as hypercholesterolemia, T2DM, coronary artery disease, and stroke, showing positive effects on endothelial function in these individuals (85). However, our results indicated the need for more evidence for these compounds in IDDM, as only two studies with B vitamins have shown positive effects on these markers (19,23).

From studies using chronic vitamin C supplementation, it was only possible to observe a positive effect on renal function by reducing the transcapillary escape rate of albumin (40). Beyond that, Davison et al. (39) showed that pre-exercise ascorbic acid can have a prophylactic effect with decreased vascular free radical generation in IDDM patients, but attention is needed to its use in this context since its antioxidant effect tends to block anabolic signaling pathways, impairing metabolic adaptations to training (86). Therefore, evidence is scarce to establish a recommendation about its supplementation.

The qualitative analysis of his review showed that vitamin E supplementation in IDDM may positively affect some markers of endothelial function, coagulation, and antioxidant defense (43-45,49-51,54,55). Although vitamin E can be related to the reduction of lipoprotein oxidation, most observational studies could not find a relationship between tocopherol plasma values and cardiovascular disease reduction in other populations (87).

In IDDM patients, glutamine supplementation should be used with caution, as the studies using this intervention observed increased nocturnal hypoglycemic events and the related mechanisms have not been elucidated (69,71). On the other hand, the use of a multi-amino acid mixture (70) and MCFA concentrate (80) could improve cognitive function during acute hypoglycemic episodes and thus be recommended as a prophylactic use in preserving brain function during these events, such as sleeping or driving, without producing high peaks of hyperglycemia (70).

The decrease in triglyceride and minor changes in HDL found in studies with omega-3 intervention included in this review (76,77,81) is in accordance with the most recent evidence of the use of this supplement (88). Based on the fact that mean baseline values of plasma lipids and lipoproteins in the samples of these studies were in the normal range and on the pathogenesis of IDDM, people with IDDM with elevated TGL levels and low HDL may benefit from omega-3 supplementation. The only study that observed a positive effect of this fatty acid on corneal neuroregeneration with the use of omega-3 was by Britten-Jones et al. (72). Additionally, the reports included in this review with a sample of pregnant women showed positive effects of omega-3 (74,75).

The data analyses performed by this review showed a significant effect of omega-3 in controlling HbA1c, but this result had some points that deserve attention: only four studies were included in the analyses, all with small samples; despite the low heterogeneity, one of the studies had a weight of 92.37% in the analysis (76); and the quality of evidence is low, according to the GRADE assessment. Due to the particularities of this meta-analysis and the low quality of evidence, there is low support for the recommendation of omega-3 supplementation in the control of HbA1c in individuals with IDDM.

Regarding the risk of bias, most of the included reports presented some concern (55.1%) or high risk (27.6%), with only ten reports with a low risk of bias (17.3%), which negatively affects the quality of data of these publications. Moreover, the GRADE assessment of the overall quality of evidence showed a ‘low' or ‘very-low' quality for all analyses in this research. Considering the high importance of the analyzed variables, it is not possible to establish recommendations based on the results of the meta-analyses, especially positive results, with vitamin D for FCP and omega-3 for HbA1c.

To the best of our knowledge, this study is the largest and most comprehensive systematic review with meta-analysis of intervention studies with nutritional supplementation in IDDM, and the only one that compiled data from different compounds in this condition and performed statistical analyses of the data. The main limitation of this review was the small number of meta-analyses and small number of studies included in those meta-analyses. This was due mainly to the underreported information in the studies, precluding meta-analyses for some biological markers, such as for endothelial function, renal function, and other variables related to the disease progression.

Even though positive results were reported in some studies, there was no consistency in findings for an indication of these compounds as adjuvant therapy in the treatment and control of complications and progression of IDDM. The dietetic intake of these compounds should be maintained as recommended for the healthy population, and clinical practice should be focused on avoiding nutritional deficiency in this population.

The current state of vitamin D research involving the control of IDDM progression in its early stage needs to be highlighted. The positive result of the meta-analysis for FCP, even with a few low-quality studies included, shows a possible effect of this vitamin in slowing down the destruction of β-cells. These results are preliminary but corroborate epidemiological findings in this population, opening the horizon for further studies and methodological standardization for the actual recommendation of vitamin D as therapy in controlling the progression of IDDM.

## Supplementary Material

Click to view [pdf].
