# Supplementary material for: Effects of dietary supplementation in treatment and control of progression and complications of insulin-dependent diabetes mellitus: a systematic review with meta-analyses of randomized clinical trials
Source: Braz J Med Biol Res. 2024 Aug 23;57:e13649. doi: 10.1590/1414-431X2024e13649 (PMC11349153; doi:10.1590/1414-431X2024e13649)
Supplement: Supplementary file 1 [file 1414-431X-bjmbr-57-e13649-suppl.pdf]

Figure S1. PRISMA 2020 Checklist. From: Page MJ, McKenzie JE, Bossuyt PM, Boutron I, Hoffmann TC, Mulrow CD, et al. The PRISMA 2020 statement: an updated guideline for reporting systematic reviews. *BMJ* 2021; 372: 71, doi: 10.1136/bmj.n71. For more information, visit: http://www.prisma-statement.org/.

Section and Topic	Item	Checklist item	Location where item is reported
**TITLE**			
Title	1	Identify the report as a systematic review.	Completed in Title
**ABSTRACT**			
Abstract	2	See the PRISMA 2020 for Abstracts checklist.	Formatted according to Nutrition Reviews guidelines
**INTRODUCTION**			
Rationale	3	Describe the rationale for the review in the context of existing knowledge.	Completed in Introduction
Objectives	4	Provide an explicit statement of the objective(s) or question(s) the review addresses.	Completed in Introduction
**METHODS**			
Eligibility criteria	5	Specify the inclusion and exclusion criteria for the review and how studies were grouped for the syntheses.	Provided in Methods
Information sources	6	Specify all databases, registers, websites, organizations, reference lists and other sources searched or consulted to identify studies. Specify the date when each source was last searched or consulted.	Provided in Methods
Search strategy	7	Present the full search strategies for all databases, registers and websites, including any filters and limits used.	Provided in Methods
Selection process	8	Specify the methods used to decide whether a study met the inclusion criteria of the review, including how many reviewers screened each record and each report retrieved, whether they worked independently, and	Provided in Methods

		if applicable, details of automation tools used in the process.	
Data collection process	9	Specify the methods used to collect data from reports, including how many reviewers collected data from each report, whether they worked independently, any processes for obtaining or confirming data from study investigators, and if applicable, details of automation tools used in the process.	Provided in Methods
Data items	10a	List and define all outcomes for which data were sought. Specify whether all results that were compatible with each outcome domain in each study were sought (e.g. for all measures, time points, analyses), and if not, the methods used to decide which results to collect.	Provided in Methods
	10b	List and define all other variables for which data were sought (e.g. participant and intervention characteristics, funding sources). Describe any assumptions made about any missing or unclear information.	Provided in Methods
Study risk of bias assessment	11	Specify the methods used to assess risk of bias in the included studies, including details of the tool(s) used, how many reviewers assessed each study and whether they worked independently, and if applicable, details of automation tools used in the process.	Provided in Methods
Effect measures	12	Specify for each outcome the effect measure(s) (e.g. risk ratio, mean difference) used in the synthesis or presentation of results.	-
Synthesis methods	13a	Describe the processes used to decide which studies were eligible for each synthesis (e.g. tabulating the study intervention characteristics and comparing against the planned groups for each synthesis (item #5)).	Provided in Methods
	13b	Describe any methods required to prepare the data for presentation or synthesis, such as handling of missing summary statistics, or data conversions.	Provided in Methods
	13c	Describe any methods used to tabulate or visually display results of individual studies and syntheses.	Provided in Methods
	13d	Describe any methods used to synthesize results and provide a rationale for the choice(s). If meta-analysis was performed, describe the model(s), method(s) to identify the presence and extent of statistical heterogeneity, and software package(s) used.	Provided in Methods
	13e	Describe any methods used to explore possible causes of heterogeneity among study results (e.g. subgroup analysis, meta-regression).	Provided in Methods
	13f	Describe any sensitivity analyses conducted to assess robustness of the synthesized results.	Provided in Methods
Reporting bias assessment	14	Describe any methods used to assess risk of bias due to missing results in a synthesis (arising from reporting biases).	Provided in Methods

Certainty assessment	15	Describe any methods used to assess certainty (or confidence) in the body of evidence for an outcome.	Provided in Methods
**RESULTS**			
Study selection	16a	Describe the results of the search and selection process, from the number of records identified in the search to the number of studies included in the review, ideally using a flow diagram.	Figure 1
	16b	Cite studies that might appear to meet the inclusion criteria, but which were excluded, and explain why they were excluded.	Specific studies were not cited however details available with the authors. Reasons for exclusion have been outlined in Figure 1 and methods
Study characteristics	17	Cite each included study and present its characteristics.	Tables S2-S4
Risk of bias in studies	18	Present assessments of risk of bias for each included study.	Tables S2-S4 and Figure S2
Results of individual studies	19	For all outcomes, present, for each study: (a) summary statistics for each group (where appropriate) and (b) an effect estimates and its precision (e.g. confidence/credible interval), ideally using structured tables or plots.	Tables S2-S4 Figures 2-3
Results of syntheses	20a	For each synthesis, briefly summarize the characteristics and risk of bias among contributing studies.	Completed in results
	20b	Present results of all statistical syntheses conducted. If meta-analysis was done, present for each the summary estimate and its precision (e.g. confidence/credible interval) and measures of statistical heterogeneity. If comparing groups, describe the direction of the effect.	Completed in results Figures 2-3

	20c	Present results of all investigations of possible causes of heterogeneity among study results.	Completed in results
	20d	Present results of all sensitivity analyses conducted to assess the robustness of the synthesized results.	-
Reporting biases	21	Present assessments of risk of bias due to missing results (arising from reporting biases) for each synthesis assessed.	-
Certainty of evidence	22	Present assessments of certainty (or confidence) in the body of evidence for each outcome assessed.	-
**DISCUSSION**			
Discussion	23a	Provide a general interpretation of the results in the context of other evidence.	Completed in discussion
	23b	Discuss any limitations of the evidence included in the review.	Completed in discussion
	23c	Discuss any limitations of the review processes used.	Completed in discussion
	23d	Discuss implications of the results for practice, policy, and future research.	Completed in discussion
**OTHER INFORMATION**			
Registration and protocol	24a	Provide registration information for the review, including register name and registration number, or state that the review was not registered.	Registered in PROSPERO
	24b	Indicate where the review protocol can be accessed, or state that a protocol was not prepared.	Provided in Abstract and Methods
	24c	Describe and explain any amendments to information provided at registration or in the protocol.	-
Support	25	Describe sources of financial or non-financial support for the review, and the role of the funders or sponsors in the review.	Declared
Competing interests	26	Declare any competing interests of review authors.	Declared
Availability of data, code and other	27	Report which of the following are publicly available and where they can be found: template data collection forms; data extracted from included studies; data used for all analyses; analytic code; any other materials used in the review.	Not provided but all information

materials			available with authors if required

Figure S2. Risk of bias assessment using the tool Risk of Bias 2 (RoB2). **D1:** Domain 1 **-** Risk of bias arising from the randomization process; **DS:** Domain S - Risk of bias due to period and transition effects (only for crossover design studies); **D2:** Domain 2 - Risk of bias due to deviations from intended interventions (effect of assignment to intervention); **D3:** Domain 3 - Risk of bias due to missing outcome data; **D4:** Domain 4 - Risk of bias in measurement of the outcome; **D5**: Domain 5 - Risk of bias in selection of the reported result; **OR:** Overall rating; NA: Not applicable. *Main study; #Follow-up report of the main study.

	**D1**	**DS**	**D2**	**D3**	**D4**	**D5**	**OR**
	**VITAMIN B COMPLEX**						
Alian (2012)	+	-	-	-	+	?	-
Chase (1990)	+	NA	+	+	+	?	?
Elbarbary (2020)	+	NA	+	+	+	+	+
Fraser (2012)	+	NA	-	-	+	+	-
Lewis (1992)	+	NA	+	+	+	?	?
Mendola (1989)	+	NA	?	+	+	?	?
Mackenzie (2006)	+	NA	+	+	+	?	?

Peña (2004)	+	+	+	+	+	?	?
Peña (2013)	+	-	+	+	+	?	?
Pozzilli (1995)	+	NA	+	+	+	?	?
Vague (1989)	+	NA	-	+	+	?	-
Valerio (1999)	+	NA	+	+	+	?	?
Wotherspoon (2008)	+	NA	-	+	+	?	-

VITAMIN D

Ataie-Jafari (2013)	+	NA	-	+	+	?	-
Bizzarri (2010)*<br>Napoli (2013)#	+	NA	?	-	+	+	-
Bogdanou (2017)	+	+	?	+	+	+	?
Gabbay (2012)	+	NA	?	+	+	?	?

Joergensen (2014)	+	NA	+	+	+	+	+
Nwosu (2021)	+	NA	+	+	+	+	+
Treiber (2015)	+	NA	+	+	+	+	+
Walter (2010)	+	NA	+	+	+	?	?

VITAMIN C

Davison (2008)	+	NA	+	+	+	+	+
Juhl (2004)	+	NA	+	+	+	?	?
Klein (1995)	+	NA	+	+	+	?	?

VITAMIN E

Astley (1999)	+	NA	-	+	+	?	-
Bursell (1999)	+	-	-	-	+	?	-
Costacou (2015)	+	+	+	+	+	+	+

Ceriello (1991)	+	NA	?	+	+	?	?
Colette (1988)	+	+	+	+	+	?	?
Duntas (1996)	+	NA	?	+	+	?	?
Economides (2005)	+	NA	-	?	+	?	-
Engelen (2005)	+	NA	+	+	+	?	?
Giannini (2017)	+	+	+	+	+	?	?
Gisinger(1988)	?	-	-	-	+	?	-
Manuel y Keenoy (2001)	+	NA	+	+	+	?	?
Parfitt (1996)	+	+	?	+	+	?	?
Pinkney (1999)	+	NA	?	+	+	?	?
Skryme-Jones (2000)* Skryme-Jones (2001)#	+	NA	+	+	+	?	?

	**VITAMIN C + VITAMIN E**						
Beckman (2003)	?	NA	+	+	+	?	?
McCance (2010)*<br>Johnston (2013)#<br>Johnston (2016)#<br>Weissgerber (2013)#	+	NA	+	+	+	+	+
	**MINERALS AND TRACE ELEMENTS**						
Jumaah (2020)	?	NA	+	+	-	?	-
Ludvigsson (2001)	+	NA	+	+	+	?	?
Shidfar (2010)	?	NA	-	+	+	?	-
	**ANTIOXIDANT COMPOUNDS**						
Andersen (1997)	+	NA	+	+	+	?	?
Huang (2008)	+	NA	+	+	+	?	?
Mollo (2012)	+	NA	?	+	+	?	?
Scaramuzza (2015)	+	NA	+	+	+	?	?

	**AMINO ACIDS**						
Mauras (2010)	+	+	+	+	+	?	?
Rossetti (2008)	+	+	+	+	+	?	?
Torres-Santiago (2017)	+	+	+	+	+	+	+
	**FATTY ACIDS**						
Britten-Jones (2021)	+	NA	+	+	+	+	+
Haines (1986)	+	NA	-	-	+	?	-
Ivanisevic (2012)* Horvaticek (2017)#	+	NA	-	-	+	+	-
Khorshidi (2022)	+	NA	-	+	+	+	-
Mori (1991)	+	NA	+	+	+	?	?
O'Mahoney (2020)	+	NA	+	+	+	+	+
Page (2009)	+	+	?	+	+	?	?

Rossing (1996)*<br>Myrup (2001)#	+	NA	-	-	+	?	-

Table S1. Strategies and terms to search for reports in the electronic databases.

**ELECTRONIC DATABASES**	STRATEGY AND TERMS FOR SEARCH
MEDLINE	#1: randomized controlled trial [pt]<br>#2: controlled clinical trial [pt]<br>#3: randomized [tiab]<br>#4: placebo [tiab]<br>#5: clinical trials as topic [mesh: noexp]<br>#6: randomly [tiab]<br>#7: trial [ti]<br>#8: #1 OR #2 OR #3 OR #4 OR #5 OR #6 OR #7<br>#9: animals [mh] NOT humans [mh]<br>#10: #8 NOT #9<br>#11: insulin-dependent diabetes mellitus AND (vitamin OR trace element OR mineral OR antioxidant OR fatty acid OR amino acid)<br>#12: #10 AND #11
Embase	#1: 'insulin-dependent diabetes mellitus'<br>#2: 'vitamin'<br>#3: 'trace element'<br>#4: 'mineral'<br>#5: 'antioxidant'<br>#6: 'fatty acid'<br>#7: 'amino acid'<br>#8: #1 AND #2<br>#9: #1 AND #3<br>#10: #1 AND #4<br>#11: #1 AND #5<br>#12: #1 AND #6<br>#13: #1 AND #7<br>#14: #8 OR #9 OR #10 OR #11 OR #12 OR #13<br>#15: #14 AND 'randomized controlled trial'/de
CENTRAL	Search performed in the "trials" section<br>#1: (insulin-dependent diabetes mellitus):ti,ab,kw<br>#2: vitamin<br>#3: trace element<br>#4: mineral<br>#5: antioxidant<br>#6: fatty acid<br>#7: amino acid<br>#8: #1 AND #2<br>#9: #1 AND #3<br>#10: #1 AND #4<br>#11: #1 AND #5<br>#12: #1 AND #6<br>#13: #1 AND #7<br>#14: #8 OR #9 OR #10 OR #11 OR #12 OR #13
LILACS	diabetes tipo 1 AND (vitamina OR elemento traço OR mineral OR antioxidante OR ácido graxo OR aminoácido)
Grey Literature Report	Insulin-dependent diabetes mellitus
ClinicalTrials.gov	Condition or disease: insulin-dependent diabetes mellitus<br>Other terms: vitamin OR mineral OR trace element OR antioxidant OR amino acid OR fatty acid<br>Study Results: "With Results"

Table S2. Characteristics of vitamin B complex, vitamin D, vitamin C, and vitamin E supplementation studies.

Author (year)	Study design	Location	Participants (gender)[a]	Duration of intervention	Diabetes duration	Age	Intervention (type)	D/F	Main outcomes	Effects	Risk of bias[b]
**Vitamin B complex**											
Alian et al. (2012) (17)	Crossover	Iran	55 (F;M)	8 w	I: 4.4 (2.1) y<br>C 3.2 (1.9) y	I: 13.2 (3.5) y<br>C: 11.5 (3.3) y	Vitamin B9<br>(folic acid)	5 mg/d	①, ③, ④	No significant changes	–
Chase et al. (1990) (18)	Parallel	USA	35 (F;M)	12 m	I: 28.7 (2.8) d<br>C: 25.3 (3.8) d<br>(mean±SE)	I: 12.5 (3.7) y<br>C: 10.8 (3.5) y	Vitamin B3<br>(NR)	≤1.5 g/d	①, ②	No significant changes	?
Elbarbary et al. (2020) (19)	Parallel	Egypt	80 (F;M)	12 s	I: 8.4 (2.4) y<br>C: 8.9 (3.0) y	I: 15.3 (1.6) y<br>C: 15.5 (1.7) y	Vitamin B1<br>(Thiamine nitrate)<br>Vitamin B6 (Pyridoxine hydrochloride)<br>Vitamin B12<br>(Cyanocobalamin)	200 mg/d<br>50 mg/d<br>1000 µg/d	①, ②, ③, ④, ⑦	↑①, ↑③, ↑④, ↑⑦	+
Fraser et al. (2012) (20)	Parallel	Norway	67 (F;M)	24 m	T: 31 y<br>(mean)	18–60 y<br>(inclusion criteria)	Vitamin B1<br>(Benfotiamine)	300 mg/d	①, ③, ④, ⑦, ⑧, ⓪<br>(nerve function)	No significant changes	–
Lewis et al. (1992) (21)	Parallel	USA	49 (F;M)	6 m	≤6 w<br>(inclusion criteria)	T: 10 (6.0) y<br>(mean±SE)	Vitamin B3<br>(nicotinamide)	40 mg/kg	①, ②	No significant changes	?
Mendola et al. (1989) (22)	Parallel	Spain	46 (F;M)	45 d	I: 60.5 (45.3) d<br>C: 74.5 (86.7) d	I: 18.3 (6.7) y<br>C: 15.5 (5.5) y	Vitamin B3<br>(nicotinamide)	1 g/d	①, ②	No significant changes	?
Mackenzie et al. (2006) (23)	Parallel	Australia	124 (F;M)	Immediate + 8 w	I1: 5.8 (3.6) y<br>I2: 5.5 (4.4) y<br>I3: 5.9 (4.0) y<br>C: 4.2 (2.8) y	I1: 14.3 (2.6) y<br>I2: 14.1 (2.6) y<br>I3: 14.3 (2.9) y<br>C: 13.6 (2.8) y	G1: Vitamin B9 (folate) + Placebo<br>G2: Placebo + Vitamin B6 (NR)<br>G3: Vitamin B9 (folate) + Vitamin B6 (NR);<br>G4: Placebo	5 mg/d<br>(folate)<br>100 mg/d<br>(Vit. B6)	③	↑③	?
Peña et al. (2004) (24)	Crossover	Australia<br>New Zealand	38 (F;M)	8 w	G1: 6.5 (3.3) y<br>G2: 6.8 (3.2) y	G1: 13.7 (2.6) y<br>G2: 13.9 (2.7) y	Vitamin B9<br>(folic acid)	5 mg/d	①, ③	No significant changes	?
Peña et al. (2013) (25)	Crossover	Australia	20 (F;M)	4 m	T: 8.1 (4.0) y	T: 15.1 (2.6) y	Vitamin B9<br>(folic acid)	0.5; 2.0; 5.0 mg/dia	①, ②, ③, ⑦	No significant changes	?
Pozzilli et al. (1995) (26)	Parallel	Italy	56 (F;M)	12 m	<4 w<br>(inclusion criteria)	I: 13.2 (6.7) y<br>C: 13.2 (6.5) y	Vitamin B3<br>(nicotinamide)	25 mg/kg/d	①, ②	No significant changes	?
Vague et al. (1989) (27)	Parallel	France	26 (F;M)	9 m	I: 28.2 (14.0) m<br>C: 26.8 (6.2) m	I: 29.8 (7.3) y<br>C: 26.8 (6.2) y	Vitamin B3<br>(nicotinamide)	3 g/d	①, ②	↑①	–
Valerio et al. (1999) (28)	Parallel	Italy	10 (F;M)	3 m	I: 5.4 (1.2) y<br>C: 3.9 (0.6) y<br>(mean±SE)	I: 11.5 (1.7) y<br>C: 11.4 (1.9) y<br>(mean±SE)	Vitamin B1<br>(lipophilic thiamine)	50 mg/d	①, ②	No significant changes	?
Wotherspoon et al (2008) (29)	Parallel	England	16 (F;M)	2 m	I: 25.6 (7.2) y<br>C:25.1 (12.0) y	I: 40.7 (9.4) y<br>C: 44.0 (12.8) y	Vitamin B9<br>(folic acid)	5 mg/d	③	No significant changes	–
**Vitamin D**											
Ataie-Jafari et al (2013) (30)	Parallel	Iran	61 (F;M)	6 m	I: 44 (14) d<br>C: 38 (18) d	I: 10.2 (2.5) y<br>C: 11.1 (1.6) y	Vitamin D (alfacalcidol)	0.25–0.50 µg/d	①, ②	No significant changes	–
Bizzarri et al. (2010) (31)	Parallel	Italy	34 (F;M)	24 m	<12 w<br>(inclusion criteria)	T: 18 (11–35) y<br>(median/min-max)	Vitamin D (calcitriol)	0.25 µg/d	①, ②	No significant changes	–

Bogdanou et al. (2017) (32)	Crossover	Germany	42 (F;M)	3 m	G1: 13.2 y<br>G2: 11.4 y<br>(mean)	G1: 48 y<br>G2: 35.5 y<br>(mean)	Vitamin D (cholecalciferol)	4000 IU/d	①, ②, ⑥	No significant changes	?
Gabbay et al. (2012) (33)	Parallel	Brazil	38 (F;M)	18 m	I: 2.2 (1.2) m<br>C: 2.7 (1.7) m	I: 13.5 (5.1) y<br>C: 12.5 (4.8) y	Vitamin D (cholecalciferol)	2000 IU/d	①, ②, ⑥, ⑧	↑⑥	?
Joergensen et al. (2014) (34)	Crossover	Denmark	48 (F;M)	12 w	40 (19–66) y<br>(median/min-max)	56 (40–74) a<br>(median/min–max)	Vitamin D (paricalcitol)	≤ 2 μg/d	①, ④	↓④	+
Napoli et al. (2013) (35)	Parallel	Italy	27 (F;M)	12 m	<12 w<br>(inclusion criteria)	I: 22.00 (2.34) y<br>C: 22.83 (2.14) y<br>(mean±SE)	Vitamin D (calcitriol)	0.25 μg/d	⓪<br>(bone metabolism)	No significant changes	–
Nwosu et al. (2021) (36)	Parallel	USA	36 (F;M)	12 m	<3 m<br>(inclusion criteria)	I: 13.25 (2.76) y<br>C: 14.28 (2.86) y	Vitamin D (ergocalciferol)	50,000 IU/w/2m + 100,000 IU/m/10m	①, ②, ⑥, ⑧	↑②, ↑⑧	+
Treiber et al. (2015) (37)	Parallel	Austria	30 (F;M)	12 m	I: 61 (20) d<br>C: 61 (28) d	I: 12 (11.0–17.5)<br>C: 13 (9.5–15.5)<br>(median/25–75%)	Vitamin D (cholecalciferol)	70 IU/kg/d	①, ②, ⑥	↑⑥	+
Walter et al. (2010) (38)	Parallel	Germany	40 (F;M)	9 m	<62 d<br>(inclusion criteria)	I: 31.4 (6.8) y<br>C: 24.0 (6.0) y<br>(median/standard deviation)	Vitamin D (calcitriol)	0.25 μg/d	①, ②	No significant changes	?
**Vitamin C**											
Davison et al. (2008) (39)	Parallel	Wales	12 (M)	Immediate	I: 14 (9.0) y<br>C: 8 (7.0) y	I: 27 (2.0) y<br>C: 27.5 (5.0) y	Vitamin C (ascorbic acid)	1g - 2h before exercise	①, ⑧	↑⑧	+
Juhl et al. (2004) (40)	Parallel	Denmark	20 (NR)	6 m	I: 18.0 (15–20) y<br>C: 18.5 (18–19) y<br>(median/25–75%)	I: 35.3 (8.5) y<br>C: 37.8 (6.6) y	Vitamin C (ascorbic acid)	6 g/dia	①, ③, ④	↑④	?
Klein et al. (1995) (41)	Parallel	Denmark	24 (M)	4 w	I: 7 (2–12) y<br>C: 8 (2–7) y<br>(mean/min–max)	I: 35 (18–38) y<br>C: 30 (19–36) y<br>(mean/min–max)	Vitamin C (ascorbic acid)	6 g/dia	③	No significant changes	?
Vitamin E											
Astley et al. (1999) (42)	Parallel	England	49 (F;M)	8 w	I: 16.0 (6.0) y<br>C: 15.1 (5.9) y	I: 38.1 (6.6) y<br>C: 38.9 (7.5) y	Vitamin E (α-tocopherol)	400 IU/d	①, ⑤, ⑦	No significant changes	–
Bursell et al. (1999) (43)	Crossover	USA	46 (F;M)	4 m	T: 4.3 (2.7) y	T: 31.6 (7.1) y	Vitamin E (NR)	1800 IU/d	①, ③, ④, ⑦, ⓪<br>(retinal variables)	↑⓪	–
Costacou et al. (2015) (44)	Parallel	USA	89 (F;M)	8 w	Hp 1-1: 36.7 (8.5) y<br>Hp 2-1: 40.1 (7.1) y<br>Hp 2-2: 40.2 (4.3) y	Hp 1-1: 49.1 (8.2) y<br>Hp 2-1: 49.3 (8.7) y<br>Hp 2-2: 50.7 (6.2) y	Vitamin E (d-α-tocopherol acetate)	400 IU/d	⑦	↑⑦	+
Ceriello et al. (1991) (45)	Parallel	Italy	30 (F;M)	2 m	I1: 6.5 (1.0) y<br>I2: 5.5 (1.4) y<br>C: 5.8 (0.7) y<br>(mean±SE)	I1: 41 (1.5) y<br>I2: 42 (1.0) y<br>C: 40 (1.3) y<br>(mean±SE)	Vitamin E (NR)	GI1: 1200 mg/d<br>GI2: 600 mg/d	①③	↑③	?
Colette et al. (1988) (46)	Crossover	France	9 (F;M)	35 d	3–33 y<br>(Time interval of treatment with insulin)	T: 53 (13.0) y	Vitamin E (NR)	1 g/d	①, ③, ⑤, ⑦	No significant changes	?
Duntas et al. (1996) (47)	Parallel	Germany	36 (F;M)	6 m	I1: 9.2 (1.4) y<br>I2: 12.4 (1.9) y<br>C: 8.7 (1.1) y	I1: 41 (3.5) y<br>I2: 45 (3.8) y<br>C: 39.5 (3.5) y	Vitamin E (d-α-tocopherol)	I1: 400 IU/d<br>I2: 800 IU/d	①, ⑦	No significant changes	?

Economides et al. (2005) (48)	Parallel	USA	32 (F;M)	12 m	T: 25 (16.0) y	T: 44 (15.0) y	Vitamin E (NR)	1.800 IU/d	③	No significant changes	–
Engelen et al. (2000) (49)	Parallel	Belgium	44 (F;M)	6 and 12 m	I: 16 (10.0) y<br>C: 16 (7.0) y	I: 42 (26–54)<br>C: 40 (23–65)<br>(median/min–max)	Vitamin E<br>(RRR-α-tocopherol)	750 IU/d	①, ⑤, ⑦	↑⑤	?
Giannini et al. (2017) (50)	Crossover	Italy	10 (F;M)	24 w	T: 12.62 (3.3) y	T: 18.87 (2.9) y	Vitamin E<br>(DL-α-tocopherol acetate)	1200 mg/d	①, ⑤, ⑦	↑⑤	?
Gisinger et al. (1988) (51)	Crossover	Austria	22 (F;M)	4 w	T: 8.4 (1.2) y	T: 23.5 (1.3) y	Vitamin E<br>(DL-α-tocopherol acetate)	400 mg/d	①, ⑤, ⑦	↑⑤	–
Manuel y Keenoy et al. (2001) (52)	Parallel	Belgium	44 (F;M)	1 y	I: 16 (10.0) y<br>C: 16 (7.0) y	I: 42 (26–54) y<br>C: 40 (23-45) y<br>(median/min-max)	Vitamin E<br>(d-α-tocopherol)	750 IU/d	⑤, ⑦	No significant changes	?
Parfitt et al. (1996) (53)	Crossover	England	12 (F;M)	8 w	T: 6.6 (6.3) y	T: 30.1 (5.0) y	Vitamin E<br>(D-α-tocopherol)	400 IU/d	①, ⑤, ⑦	No significant changes	?
Pinkney et al. (1999) (54)	Parallel	England	46 (F;M)	3 m	T: 16.0 (9.3–24.0) y<br>(median/25–75%)	T: 39.0 (10.8) y	Vitamin E<br>(d-α-tocopherol)	500 IU/d	①, ③, ④, ⑤	↑③	?
Skyrme-Jones et al. (2000) (55)	Parallel	Australia	41 (F;M)	3 m	I: 110 (71.0) m<br>C: 119 (60.0) m	I: 23 (6.0) y<br>C: 28 (5.0) y	Vitamin E<br>(all-rac-α-tocopherol)	1000 IU/d	③, ⑤	↑③, ↑⑤	?
Skyrme-Jones et al. (2001) (56)	Parallel	Australia	41 (F;M)	3 m	I: 110 (71.0.) m<br>C: 119 (60.0) m	T: 25.8 (5.0) y	Vitamin E<br>(all-rac-α-tocopherol)	1000 IU/d	③	No significant changes	?
**Vitamin C + Vitamin E**											
Beckman et al. (2003) (57)	Parallel	USA	26 (F;M)	180 d	T: 11.8 (2.8) y	36 (10.0) y	Vitamin C<br>(ascorbate)<br>Vitamin E<br>(α-tocopherol)	1000 mg/d<br>400 IU/d	③, ⑤	No significant changes	?
Johnston et al. (2013) (58)	Parallel	Northern Ireland	57 (F)	Beginning at the 8th to the 22nd GA until delivery	I: 11.99 (8.5) y<br>C: 17.20 (8.9) y	I: 31.12 (5.0) y<br>C: 30.85 (4.9) y	Vitamin C (NR)<br>Vitamin E (NR)	1000 mg/d<br>400 IU/d	⑤	No significant changes	+
Johnston et al. (2016) (59)	Parallel	Northern Ireland	57 (F)	Beginning at the 8th to the 22nd GA until delivery	I: 12 (8.5) y<br>C: 17 (8.9) y	I: 31 (5.0) y<br>C: 31 (4.9) y	Vitamin C (NR)<br>Vitamin E (NR)	1000 mg/d<br>400 IU/d	⑤	No significant changes	+
McCance et al. (2010) (60)	Parallel	Northern Ireland<br>Scotland<br>England	762 (F)	Beginning at the 8th to the 22nd GA until delivery	I: 14.0 (8.3) y<br>C: 15.0 (8.0) y	I: 29.5 (5.6) y<br>C: 29.6 (5.7) y	Vitamin C (NR)<br>Vitamin E (α-tocopherol)	1000 mg/d<br>400 IU/d	⓪<br>(maternal and newborn parameters)	No significant changes	+
Weissgerber et al. (2013) (61)	Parallel	Northern Ireland<br>Scotland<br>England	762 (F)	Beginning at the 8th to the 22nd GA until delivery	Hp 1-1: 14.6 (7.9) y<br>Hp 2-1: 14.1 (8.0) y<br>Hp 2-2: 15.1 (8.5) y	Hp 1-1: 29.7 (5.7) y<br>Hp 2-1: 29.6 (5.5) y<br>Hp 2-2: 29.8 (5.6) y	Vitamin C (NR)<br>Vitamin E<br>(α-tocopherol)	1000 mg/d<br>+<br>400 IU/d	⓪<br>(maternal and newborn parameters)	No significant changes	+

Data are reported as means±SD or in parentheses as reported in the study. [a]Total IDDM randomized. [b]RoB 2 overall rating: + low risk; ?: some concern; –: high risk. D/F: dose/frequency; M: male; F: female; C: control; I: intervention; T: total (C+I); G: group; d: days; w: weeks; m: months; y: years; GA: gestational age; NR: not reported. ① glycemic control, ② β-cell activity, ③ endothelial and coagulation function, ④ renal function, ⑤ antioxidant defense, ⑥ immunological profile, ⑦ lipid profile, ⑧ inflammatory markers, ⑨ cognitive function, ⓪ others.

Table S3. Characteristic of the included mineral, trace elements, and antioxidant compounds supplementation studies.

Author (year)	Study design	Location	Participants (gender)[a]	Duration of intervention	Diabetes duration	Age	Intervention (type)	D/F	Main outcomes	Effects	Risk of bias[b]
**Minerals and trace elements**											
Jumaah et al. (2020) (62)	Parallel	Iraq	50 (F;M)	180 d	NR	1–16 y (inclusion criteria)	Vitamin D (cholecalciferol) Magnesium (NR) Zinc (NR)	800 IU/d 3 mg/kg/d 2 mg/d	⓪ (Symptoms and signs of IDDM and ketoacidosis)	↑⓪	–
Ludvigsson et al. (2001) (63)	Parallel	Sweden	46 (F;M)	2 y	Included at the time of the first insulin dose	I: 10.2 (3.0) y C: 10.5 (3.0) y	Vitamin B3 (nicotinamide) Vitamin C (NR) Vitamin E (NR) β-carotene Selenium (NR)	300–900 mg/d 300–900 mg/d 30–90 mg/d 5–15 mg/d 150–450 µg/d	①, ②, ⑥	No significant changes	?
Shidfar et al. (2010) (64)	Parallel	Iran	52 (F;M)	3 m	I: 3.86 (1.9) y C: 4.10 (2.2) y	I: 13.16 (3.4) y C: 13.56 (3.5) y	Vitamin A (retinyl palmitate) Zinc (elemental zinc)	12.500 UI/alternate days 10 mg/d	①, ⑦	↑⑦	–
**Antioxidant compounds**											
Andersen et al. (1997) (65)	Parallel	Denmark	34 (F;M)	12 w	I: 15.9 (1.8) y C: 20.8 (2.5) y	I: 35.0 (2.0) y C: 35.3 (2.4) y	Coenzime Q10	100 mg/d	①, ②, ⑦	No significant changes	?
Huang et al. (2008) (66)	Parallel	USA	40 (F;M)	3 m	I: 7 (3.5) y C: 7 (4.1) y	I: 14 (2.4) y C: 15 (1.9) y	ALA (Controlled-release α-lipoic acid)	600–1200 mg/d	①, ⑤	No significant changes	?
Mollo et al. (2012) (67)	Parallel	Italy	51 (F;M)	5 w	NR	I: 43 (9) y C: 46 (11) y	ALA (α-lipoic acid)	600 mg/d	③	No significant changes	?
Scaramuzza et al. (2015) (68)	Parallel	Italy	71 (F;M)	6 m	I: 7.7 (4.9) y C1: 8.2 (5.6) y C2: 8.8 (5.7) y	I: 16.1 (3.1) y C1: 16.0 (3.4) y C2: 16.5 (4.3) y	ALA (Controlled-release α-lipoic acid)	400 mg/d	①, ②, ③	No significant changes	?

Data are reported as means±SD or in parentheses as reported in the study. [a]Total IDDM randomized. [b]RoB 2 overall rating: +: low risk; ?: some concern; –: high risk. D/F: dose/frequency; M: male; F: female; C: control; I: intervention; T: total (C+I); G: group; d: days; w: weeks; m: months; y: years; GA: gestational age; NR: not reported. ① glycemic control, ② β-cell activity, ③ endothelial and coagulation function, ④ renal function, ⑤ antioxidant defense, ⑥ immunological profile, ⑦ lipid profile, ⑧ inflammatory markers, ⑨ cognitive function, ⓪ others.

Table S4. Characteristics of the included amino acid and fatty acid supplementation studies.

Author (year)	Study design	Location	Participants (gender)[a]	Duration of intervention	Diabetes duration	Age	Intervention (type)	D/F	Main outcomes	Effects	Risk of bias[b]
**Amino acids**											
Mauras et al. (2010) (69)	Crossover	USA	10 (F;M)	Immediate	T: 6.2 (3.3) y	T: 15.2 (1.4) y	Glutamine	0.25 g/kg pre-exercise 0.25 g/kg at bedtime	①	↑①	?
Rossetti et al. (2008) (70)	Crossover	Italy	10 (F;M)	Immediate	T: 17 (7.8) y	T: 30 (8) y	Amino acid mixture	42g	⑨	↑⑧	?
Torres-Santiago et al. (2017) (71)	Crossover	USA	13 (F;M)	Immediate	T: 7.9 (1.3) y	T: 15.9 (1.6) y	Glutamine	0.25 g/kg pre-exercise 0.25 g/kg at bedtime	①	↑①	+
**Fatty acids**											
Britten-Jones et al. (2021) (72)	Parallel	Australia	43 (F;M)	180 d	I: 14.0 (6.0–25.0) y<br>C: 16.5 (7.0–26.0) y<br>(median/25–75%)	I: 48.1 (19.2) y<br>C: 40.5 (19.6) y	Omega-3 (fish oil)	EPA: 1080 mg/d<br>DHA: 720 mg/d	①, ⑦, ⓪ (nerve function and retinal variables)	↑⓪	+
Haines et al. (1986) (73)	Parallel	England	41 (F;M)	6 w	NR	I: 43.2 (9.0) y<br>C: 41.6 (10.0) y	Omega-3 (fish oil)	EPA: 2.7 mg/d<br>DHA: 1.9 mg/d	①, ③, ⑦	↑③	–
Horvaticek et al. (2017) (74)	Parallel	Croatia	111 (F)	Beginning of the 9th SG until delivery	5-30 y (inclusion criteria)	I: 29.8 (5.5) y<br>C: 29.6 (5.8) y	Omega-3 (fish oil)	EPA: 120 mg/d<br>DHA: 616 mg/d	⓪ (maternal and newborn parameters)	↑⓪	–
Ivanisevic et al. (2021) (75)	Parallel	Croatia	111 (F)	Beginning of the 11th to 12th SG until delivery	I: 11.8 (6.9) y<br>C: 13.8 (7.3) y	I: 29.4 (5.3) y<br>C: 30.5 (5.2) y	Omega-3 (fish oil)	EPA: 120 mg/d<br>DHA: 616 mg/d	⓪ (maternal and newborn parameters)	↑⓪	–
Khorshidi et al. (2022) (76)	Parallel	Iran	60 (F;M)	12 w	I: 7.6 (2.3) y<br>C: 7.4 (2.8) y	I: 13.8 (2.3) y<br>C: 12.9 (2.4) y	Omega-3 (NR)	EPA: 180 mg/d<br>DHA: 120 mg/d	①, ③, ④, ⑦, ⓪ (maternal and newborn parameters)	↑③, ↑⑦	–
Mori et al. (1991) (77)	Parallel	Australia	27 (M)	3 w	3–34 y (min–max)	I: 34.4 (1.9) y<br>C1: 33.8 (2.1) y<br>C2: 34.2 (1.9) y<br>(mean±SE)	Omega-3 (fish oil)	ω-3: 5.2g<br>ω-6: 0.3g	⑦	↑⑦	?
Myrup et al. (2001) (78)	Parallel	Denmark	36 (F;M)	12 m	I: 20 (4) y<br>C: 20 (6) y	I: 32 (7) y<br>C: 34 (10) y	Omega-3 (cod liver oil)	EPA: 2 g/d<br>DHA: 2.6 g/d	③, ④	No significant changes	–
O'Mahoney et al. (2020) (79)	Parallel	England	27 (F;M)	6 m	I: 15 (13) y<br>C: 21 (12) y	I: 32 (12) y<br>C: 36 (17) y	Omega-3 (cod liver oil)	EPA: 2.3 g/d<br>DHA: 0.8 g/d	①, ③, ⑦, ⑧, ⓪ (maternal and newborn parameters)	No significant changes	+
Page et al. (2009) (80)	Crossover	USA	11 (F;M)	Immediate	T: 15.9 (9.5) y	T: 34.8 (8.9) y	Medium Chain Fatty Acid (coconut oil)	40g	⑨	↑⑨	?
Rossing et al. (1996) (81)	Parallel	Denmark	36 (F;M)	12 m	I: 20 (4) y<br>C: 20 (6) y	I: 32 (7) y<br>C: 34 (10) y	Omega-3 (cod liver oil)	EPA: 2.0 g/d<br>DHA: 2.6 g/d	①, ④, ⑦	↑⑦	–

Data are reported as means±SD or in parentheses as reported in the study. [a]Total IDDM randomized. [b]RoB 2 overall rating: +: low risk; ?: some concern; –: high risk. DI: duration of intervention; D/F: dose/frequency; M: male; F: female; C: control; I: intervention; T: total (C+I); G: group; d: days; w: weeks; m: months; y: years; GA: gestational age; NR: not reported. ① glycemic control, ② β-cell activity, ③ endothelial and coagulation function, ④ renal function, ⑤ antioxidant defense, ⑥ immunological profile, ⑦ lipid profile, ⑧ inflammatory markers, ⑨ cognitive function, ⓪ others.

Table S5. GRADE summary of findings for the assessment of quality of evidence.

Question: Effects of nutritional compounds supplementation compared to placebo on markers of glucose metabolism in individuals with insulin-dependent diabetes mellitus											
Quality assessment							No of patients		Effect	Quality	Importance
No. of studies	Study design	Risk of bias	Inconsistency	Indirectness	Imprecision	Other considerations	Intervention	Placebo	Absolute (95%CI)		
Vitamin B3											
HbA1c											
3	RCT	serious[a]	serious[b]	not serious	not serious	N/A	57	57	RE: –1.01 (–2.57, 0.44)	VERY LOW[e]	CRITIC
Fasting C-peptide											
3	RCT	serious[a]	not serious	not serious	serious[d]	N/A	56	55	RE: 0.04 (–0.02, 0.10)	VERY LOW[f]	IMPORTANT
Insulin dose requirement											
3	RCT	serious[a]	not serious	not serious	not serious	N/A	55	55	RE: –0.20 (–1.33, 0.18)	LOW[g]	IMPORTANT
Vitamin D											
HbA1c											
4	RCT	serious[a]	not serious	serious[c]	not serious	N/A	86	79	RE: -0.23 (–0.53, 0.08)	VERY LOW[h]	CRITIC
Fasting C-peptide											
4	RCT	serious[a]	not serious	serious[c]	serious[d]	N/A	71	69	RE: 0.05 (0.01, 0.09)	VERY LOW[i]	IMPORTANT
Vitamin E											
HbA1c											
4	RCT	serious[a]	not serious	not serious	not serious	N/A	75	52	RE: -0.26 (–0.56, 0.05)	LOW[g]	CRITIC
Omega-3											
HbA1c											
4	RCT	serious[a]	not serious	not serious	not serious	N/A	71	69	RE: -0.33 (–0.54, –0.12)	LOW[g]	CRITIC

RCT: Randomized controlled trial; CI: confidence interval; RE: random effect; N/A: not applicable. [a]There is a serious risk of bias because more than half of the studies included in the analysis were rated as having "some concern" or "high risk" by Risk of Bias 2 (RoB2). [b]There is a serious risk of inconsistency due to the high heterogeneity of the analysis. [c]There is a serious risk of indirect evidence due to the different presentation forms of the compounds. [d]There is a high risk of imprecision because the sample size of the analysis is smaller than the calculated OIS. [e]Very low quality of evidence due to serious risk of bias and inconsistency. [f]Very low quality of evidence due to serious risk of bias and imprecision. [g]Low quality of evidence due to serious risk of bias. [h]Very low quality of evidence due to serious risk of bias and indirectness. [i]Very low quality of evidence due to serious risk of bias, indirectness, and imprecision.
